# Visual Attention to Suffering After Compassion Training Is Associated With Decreased Amygdala Responses

**DOI:** 10.3389/fpsyg.2018.00771

**Published:** 2018-05-22

**Authors:** Helen Y. Weng, Regina C. Lapate, Diane E. Stodola, Gregory M. Rogers, Richard J. Davidson

**Affiliations:** ^1^Osher Center for Integrative Medicine, University of California, San Francisco, San Francisco, CA, United States; ^2^Department of Psychiatry, University of California, San Francisco, San Francisco, CA, United States; ^3^Center for Healthy Minds, University of Wisconsin-Madison, Madison, WI, United States; ^4^Helen Wills Neuroscience Institute, University of California, Berkeley, Berkeley, CA, United States; ^5^Department of Psychiatry, University of Wisconsin-Madison, Madison, WI, United States

**Keywords:** compassion, meditation, visual attention, amygdala, functional magnetic resonance imaging (fMRI)

## Abstract

Compassion meditation training is hypothesized to increase the motivational salience of cues of suffering, while also enhancing equanimous attention and decreasing emotional reactivity to suffering. However, it is currently unknown how compassion meditation impacts visual attention to suffering, and how this impacts neural activation in regions associated with motivational salience as well as aversive responses, such as the amygdala. Healthy adults were randomized to 2 weeks of compassion or reappraisal training. We measured BOLD fMRI responses before and after training while participants actively engaged in their assigned training to images depicting human suffering or non-suffering. Eye-tracking data were recorded concurrently, and we computed looking time for socially and emotionally evocative areas of the images, and calculated visual preference for suffering vs. non-suffering. Increases in visual preference for suffering due to compassion training were associated with decreases in the amygdala, a brain region involved in negative valence, arousal, and physiological responses typical of fear and anxiety states. This pattern was specifically in the compassion group, and was not found in the reappraisal group. In addition, compassion training-related increases in visual preference for suffering were also associated with decreases in regions sensitive to valence and empathic distress, spanning the anterior insula and orbitofrontal cortex (while the reappraisal group showed the opposite effect). Examining visual attention alone demonstrated that engaging in compassion in general (across both time points) resulted in visual attention preference for suffering compared to engaging in reappraisal. Collectively, these findings suggest that compassion meditation may cultivate visual preference for suffering while attenuating neural responses in regions typically associated with aversive processing of negative stimuli, which may cultivate a more equanimous and nonreactive form of attention to stimuli of suffering.

## Introduction

Compassion involves affective, cognitive, as well as motivational responses to suffering, where the person who witnesses suffering feels a sense of caring for and wanting to help others experiencing suffering (Goetz et al., 2010; Dahl et al., 2015; Engen and Singer, 2016). Compassion may be cultivated through mental training such as meditation (Salzberg, 1997), and compassion meditation has been shown to enhance positive emotional responses (Fredrickson et al., 2008; Hutcherson et al., 2008; Klimecki et al., 2012, 2014) and prosocial behavioral responses to suffering (Leiberg et al., 2011; Condon et al., 2013; Weng et al., 2013, 2015; McCall et al., 2014; Ashar et al., 2016). However, little is known about the more basic attentional mechanisms that may support this affective and behavioral profile due to compassion meditation training.

Compassion increases the motivational salience of cues of suffering, where attention is drawn to suffering due to the motivation to care for others’ well-being and the desire to relieve suffering (Goetz et al., 2010). Attentional qualities which support compassion are thought to be focused, sustained, and selective to cues of suffering (Halifax, 2012). Compassion is also an embodied dynamic process (Halifax, 2012), which may include greater visual attention to cues of suffering from the observer to the receiver of compassion. Indeed, compassion often involves gazing and leaning towards others (Eisenberg et al., 1988; Gurthrie et al., 1997), and gaze is typically directed towards stimuli that are consistent with goals (Isaacowitz, 2006). In particular, compassionate goals often involve understanding and caring for others who are suffering; therefore, more visual attention should be deployed to stimuli of suffering to perceive and understand the nature of suffering, and possibly to motivate prosocial action (Davidson and Harrington, 2001; Goetz et al., 2010). This may result in a visual preference for cues of suffering compared to neutral cues. For example, if someone is injured in a crowd of people, the observer would need to look at cues of suffering such as the site of the injury and the person’s facial expressions, and disengage from neutral cues such as non-injured areas and faces of other people who are not in pain, in order to engage in targeted helping behaviors such as dressing the wound. This is in contrast to other emotion regulatory strategies that have different motivational goals such as cognitive reappraisal (Jackson et al., 2000; van Reekum et al., 2007; Ochsner and Gross, 2008; Lee et al., 2009), where meaning of stimuli is cognitively reinterpreted, and may direct visual attention *away* from stimuli of suffering to aid the goal of reducing negative emotion. Indeed, decreased emotional responses and neural changes during cognitive reappraisal is explained in part by a reduction in the degree to which people look at emotionally evocative parts of negative images (van Reekum et al., 2007).

Compassion meditation involves mental exercises that cultivate concern for and motivation to relieve suffering of the self and others (Salzberg, 1997; Hofmann et al., 2011). For example, in Weng et al. (2013), participants cultivated compassion for a loved one, the self, a stranger, and a difficult person (someone with whom there is conflict). For each target of compassion meditation, three steps were practiced: (1) *envisioning suffering*, or imagining a time each person has suffered; (2) *mindful attention to reactions to suffering*, where non-judgmental attention is brought to sensations, thoughts, and feelings that arise in response to envisioning suffering; and (3) *cultivating compassion*, where feelings of care and concern for the target are practiced as well as a desire to relieve suffering (Weng et al., 2013, 2017). These practices are thought to enhance prosocial action through emotion regulatory pathways (Weng et al., 2013, 2017) of (1) reducing empathic distress (also called personal distress), which involves aversive physiological, emotional, and cognitive reactions to others’ suffering (Batson, 1991; Ashar et al., 2016), and (2) enhancing compassion or empathic concern, which involve care for and desire to alleviate others’ suffering (Batson, 1991; Goetz et al., 2010).

Previous studies have demonstrated that practicing compassion meditation internally results in increased prosocial behavior externally towards strangers in novel situations including altruistic monetary giving (Weng et al., 2013; McCall et al., 2014; Ashar et al., 2016), costly and non-costly helping in a computer game (Leiberg et al., 2011), and offering a seat to an injured stranger (Condon et al., 2013). However, less has been done to investigate intermediate steps that may lead to prosocial behavior, such as changes in visual attention. Because visual imagery and visual perception recruit largely overlapping brain regions (Ganis et al., 2004), it is plausible that internal visual imagery in compassion meditation may *transfer* to external visual perception and increase attention given to cues of suffering. In addition, because compassion meditation aims to cultivate a more balanced non-judgmental response to suffering, it is possible that while visual attention to suffering may increase motivated by empathic concern, emotional responses may become less negative and reactive (Weng et al., 2017), which should decrease neural and physiological responses that could induce empathic distress, motivate self-focus, and result in withdrawal (Batson et al., 1987; Batson, 1991).

Greater visual engagement with stimuli of suffering in compassion may require regulation of emotional responses to suffering. Neurally, emotional responses to negative stimuli are often correlated with increased amygdala activation, which can be measured using functional magnetic resonance imaging (fMRI) (Zald, 2003; Phelps and LeDoux, 2005). The amygdala has been consistently shown to be activated in response to aversive stimuli (Zald, 2003), including visual stimuli depicting human emotional and physical suffering (Bruneau et al., 2015). In addition, subregions of amygdala such as the centromedial nucleus of the amygdala have direct projections to brain regions such as the lateral hypothalamus, basal forebrain, and brainstem, which mediate physiological responses associated with emotions via activation of the sympathetic nervous system (Davis and Whalen, 2001; Phelps and LeDoux, 2005). This includes physiological responses typical of fear and anxiety (Phelps and LeDoux, 2005) and arousal responses to social stimuli (Davis et al., 2010), which may motivate self-focus and withdrawal, rather than prosocial engagement (Batson, 1991). In the context of witnessing another’s suffering, the amygdala may be activated due to empathic distress, particularly if increased visual engagement with suffering is occurring. Compassion meditation training may therefore *reduce* activation of the amygdala in response to suffering while actively engaging in compassion, reflecting more equanimous emotional responses (Desbordes et al., 2014) which may decrease activation of the sympathetic nervous system.

Recent understandings of amygdala functioning suggest that it has a more general function in processing motivational salience, which can include stimuli that are negative, positive, arousing, interesting, or unusual, depending on a perceiver’s goals (Adolphs et al., 1999; Hamann et al., 2002; Phan et al., 2003; Cunningham and Brosch, 2012). Engaging in compassion should increase the motivational salience of suffering, which may activate the amygdala and stimulate physiological output to indicate the presence of suffering, which can then facilitate the recruitment of prosocial responses. In this case, it is possible that compassion meditation training may increase amygdala activation as a reflection of greater salience of cues of suffering and motivation to relieve suffering. Supporting this interpretation, Desbordes et al. (2012) found increased amygdala activation after compassion training while attending to negative visual stimuli (i.e., not actively engaging in compassion). Furthermore, the level of amygdala activation was negatively correlated with depression symptoms, suggesting functional utility in greater amygdala activation to negative stimuli due to compassion training.

While our previous work demonstrated that short-term compassion meditation training increased prosocial behavior compared to reappraisal training (Weng et al., 2013), whether increased visual attention to cues of suffering due to compassion training would be associated with increased or decreased amygdala activation is unknown and is the subject of this report. Here, in a subset of participants from a previously published dataset (Weng et al., 2013), we conducted a preliminary investigation of whether compassion meditation training compared to cognitive reappraisal training would result in changes in visual attention to cues of human suffering while actively engaging in regulatory strategies, and of how these changes in visual attention were associated with neural responses to suffering. In our study, reappraisal training served as an active control training condition where emotions are regulated for a different motivational goal, which is more *self*-oriented to decrease personal negative emotions. This is in contrast to compassion, which is more *other*-oriented to enhance prosocial motivation and action. Therefore, individual differences in visual preference to suffering due to the different trainings may reflect different motivational goals, which could result in differential associations with responses indexed by amygdala activation.

Participants viewed images of human suffering and non-suffering before and after training in the fMRI scanner, and eye-tracking data were collected during the brain scans. Visual preference for suffering was determined by the percentage looking time within regions of images depicting social and affective cues of suffering vs. non-suffering. Using an individual differences framework, we tested whether compassion training-related increases in visual preference for suffering would be differentially associated with amygdala activation compared to reappraisal training. In addition, we investigated the direction of the relationship due to compassion training, testing whether greater visual preference for suffering would be associated with (a) reduced neural responses in the amygdala (supporting the empathic distress interpretation of amygdala engagement), or (b) increased amygdala responses (supporting the motivational salience interpretation of amygdala engagement). In addition, we tested the impact of compassion vs. reappraisal training on visual attention to suffering, independent of its relationship with neural responses. Lastly, in preliminary exploratory analyses, we investigated whether greater visual preference for suffering following compassion training may reflect prosocial motives and be associated with increases in prosocial behavior in the Redistribution Game, an economic exchange paradigm (previously reported in this sample, Weng et al., 2013).

## Materials and Methods

### Participants

Fifty-six participants completed the entire protocol, which included being randomized to compassion or reappraisal training, completing 2 weeks of 30 min daily training (11/14 practice days were required), and attending a functional magnetic resonance imaging (fMRI) session before and after training. Participants were healthy adults (18–45 years of age), MRI-compatible, right-handed, and had no previous experience in meditation or cognitive-behavioral therapy. Data analyses were conducted on a subset of the sample who had adequate pupil registration and eye-tracking data at both pre- and post-training (see section “Materials and Methods”; total *N* = 24; Compassion *n* = 12, mean age = 20.92 years, SD = 2.19, female *n* = 7, White *n* = 12; Hispanic or Latino *n* = 1; Reappraisal *n* = 12, mean age = 21.58 years, SD = 2.87, female *n* = 8, Asian *n* = 1, White *n* = 10, Black or African American *n* = 1). The groups did not differ in age, gender, baseline trait empathic concern (Davis, 1980), or practice time (all *p*s > 0.24). The experiment was approved by the University of Wisconsin-Madison Health Sciences Institutional Review Board, all procedures were performed in accordance with these guidelines, and all subjects gave informed consent and were paid for participation.

### Procedure

#### Overview

Participants came to the laboratory on three occasions. At Visit 1, they were randomized to compassion or reappraisal training where they were briefly instructed in the assigned strategy and practiced the fMRI task in a mock MRI scanner. Visit 2 occurred approximately 1 week later, where they completed the pre-training fMRI scan, and began training later that day. Visit 3 occurred immediately after the 2 weeks of training was completed, and included the post-training fMRI scan and the altruistic behavior task (outside of the scanner). Eye-tracking data were collected during the fMRI scans.

### Trainings

Training consisted of practicing compassion meditation or reappraisal using guided audio instructions via the Internet or compact disc for 30 min/day for 2 weeks. Compassion trainees practiced cultivating feelings of compassion for different targets including a loved one, the self, a stranger, and a difficult person (someone with whom they had conflict). For each person, they imagined a time when the person had suffered, brought non-judgmental and balanced attention to reactions to suffering, and then practiced wishing the person relief from suffering. They repeated compassion-generating phrases such as, “May you be free from suffering. May you have joy and happiness.” They were also instructed to pay attention to bodily sensations (particularly around the heart) and to envision a golden light extending from their heart to the heart of the other person.

The reappraisal trainees practiced re-interpreting personally stressful events to decrease negative affect. They practiced the 3 strategies in response to daily stressors such as having an argument with a significant other. Strategies included: (1) thinking about the situation from a different perspective (such as thinking that the argument was helpful in working through conflict), (2) thinking about the situation from a friend or family member’s perspective, and (3) imagining a year had gone by and a positive outcome had occurred. Reappraisal training used common approaches in cognitive-behavioral therapy and was designed by a licensed clinical psychologist. Participants were required to complete at least 11/14 trainings and training adherence was monitored by the study team. Self-report ratings from daily reports of trainings showed that compassion trainees reported increased compassion for most targets after each session, and reappraisal trainees reported decreased negative emotions after each session. See Weng et al. (2013) for full training details and download trainings at https://centerhealthyminds.org/well-being-tools/compassion-training/.

### Study Measures

#### Functional Magnetic Resonance Imaging (fMRI)

##### fMRI data acquisition

Whole-brain functional and anatomical images were acquired using a General Electric 3 Tesla MRI scanner (GE Medical Systems, Waukesha, WI, United States) with LX software (version ESE12M4), a transmit-receive quadrature birdcage coil, and Nvi (40 mT/m; 150 mT⋅m^-1^⋅ms^-1^ slew rate) gradients. Functional images were acquired using a T2^∗^-weighted gradient-echo, echo planar imaging (EPI) pulse sequence [30 sagittal slices, 4 mm thickness, 1 mm interslice gap; 64 × 64 matrix; 240 mm field of view (FOV); repetition time (TR)/echo time (TE)/Flip, 2000 ms/30 ms/90o; 146 whole-brain volumes per block]. A high-resolution T1- weighted anatomical image was also acquired (T1-weighted inversion recovery fast gradient echo; 256 × 256 in-plane resolution; 240 mm FOV; 124 × 1.2 mm axial slices).

##### fMRI task paradigm

The fMRI task and eye-tracking data were collected before and after training. Participants employed their assigned emotion regulation strategy to a series of images depicting human suffering (Negative) or non-suffering (Neutral) from the International Affective Picture System (Lang et al., 1999) (800 × 600 resolution). Compassion trainees were instructed to evoke feelings of compassion while silently repeating compassion-generating phrases. In contrast, Reappraisal trainees were instructed to decrease negative emotions by silently re-interpreting the emotional meaning of the images (such as, “This is a scene from a movie, and this person is not really injured”). Strategies included thinking of a better outcome and viewing the scene as fake or not real, which are similar reappraisal strategies as previously reported in the literature (Jackson et al., 2000; van Reekum et al., 2007; Ochsner and Gross, 2008; Lee et al., 2009). Negative images depicted emotional distress, physical pain, or acts of violence (e.g., burn victim, crying child). Neutral images depicted people in non-emotional situations, such as working or walking on a street. Two parallel sets of images (20 negative and 16 neutral) were created to ensure that participants viewed different images before and after training. Set order was counterbalanced and randomized. Images were pseudorandomized so that 3 or more images from either condition were not presented in a row. Image randomization was performed once for each set and then fixed. Images were balanced across sets for published normative ratings of valence and arousal (Lang et al., 1999), as well as stimulus properties of hue, luminance, and saturation (all *p*s > 0.1).

Participants were instructed to view the images and regulate their emotional responses for each 12-s image presentation over 3 blocks. Each block included: (a) fixation baseline (20 s), (b) auditory and visual instruction (“Compassion” or “Reappraisal”; 3 s), (c) fixation cross (5–7 s), (d) 12 cycles of image presentation (Negative or Neutral; 12 s) and fixation inter-trial interval (5–11 s, randomized), and (f) final baseline (17–38 s). Three Regulation blocks were intermixed with three Attend blocks, where participants did not change their emotional response. Because neural activation was related to prosocial outcomes only during Regulation and not Attend trials (Weng et al., 2013, 2017), we primarily focused our analyses on Regulation trials, and conducted follow up analyses in the Attend condition.

##### fMRI pre-processing

Image analysis was performed with AFNI (Cox, 1996) unless otherwise noted. Data were slice-time corrected and motion corrected with realignment to the first volume. They were then field map corrected (Jezzard and Clare, 1999) using prelude (Smith et al., 2004) from FSL and in-house software^1^, and spatially smoothed using a Gaussian kernel with a full width at half maximum of 6 mm. Anatomical images from post-training were first normalized to the Montreal Neurological Institute (MNI152) template using an affine transformation with FLIRT (Jenkinson et al., 2002), and then re-normalized to the MNI152 template using a non-linear algorithm implemented by FNIRT in FSL. The resulting warp matrices were applied to the functional data (re-sampled to 2 mm^3^).

##### fMRI first level (subject) analysis

Functional magnetic resonance imaging analyses were performed on participants with adequate pupil gaze registration at both pre- and post-training (*n* = 24). Functional data were modeled with a general linear model (GLM) in AFNI (Cox, 1996), and the hemodynamic response was modeled with a block function for the full 12-s trial period. The GLM modeled hemodynamic responses to all conditions in the study (Regulation: Regulate or Attend, Valence: Negative or Neutral), and activation to Regulate trials were analyzed for this study. For each participant, we computed a Negative–Neutral contrast, which was interrogated with the eye-tracking data. Regressor betas from the Negative–Neutral contrast were converted to percent signal change (PSC). PSC maps were created for each time point (Pre and Post) and were normalized to the MNI152 2 mm template, which were used to compute a Post–Pre training difference PSC map, as well as an average PSC map across both time points.

#### Eye-Tracking Data

Visual attention was assessed during the fMRI experiment using an eye-tracking iView X system (v. 1.3.31) with a remote eye-tracking device (SensoMotoric Instruments, Teltow, Germany), which was interfaced with the fiber optic goggle system while participants performed the fMRI task. The iView system enables the simultaneous display of (1) video monitoring of eye movements (which allows us to monitor visual engagement and sleepiness) and (2) tracking over time of pupil gaze position over the 800 × 600 field of view. Prior to the start of the first scan, gaze fixations were calibrated by asking the participant to focus their attention on each of 9 dots presented in a random order in either one of the 4 corners of the display space, midway between each corner, and in the middle of the screen. Movement of the pupil was monitored at 60 Hz during the entire fMRI experiment. Data quality was assessed by observing the iView display for congruence between video monitoring of eye movements and analogous tracking of pupil gaze movements in the field of view. Participants with missing data at any time point due to technical issues (malfunctioning eye-tracking device, computer, or e-prime errors) were excluded (*n* = 12). Participants that demonstrated poor pupil registration (due to poor initial registration or interference of signal such as excessive blinking, which resulted in flickering and inconsistent signal that moved outside the field of view, or mismatch between video monitoring of eye position and displayed pupil position) were also not included for analyses (*n* = 20). Adequate eye tracking data by these standards were acquired from 24 participants at both pre- and post-training (Compassion *n* = 12, Reappraisal *n* = 12). Those with adequate data at both time points (*n* = 24) did not significantly differ from those who did not (*n* = 32) in age, gender, group assignment, baseline empathic concern (Davis, 1980), average bilateral amygdala activation (at Pre, Post, or Post–Pre training), or ranked redistribution behavior (all *p*s > 0.11).

**FIGURE 1:**
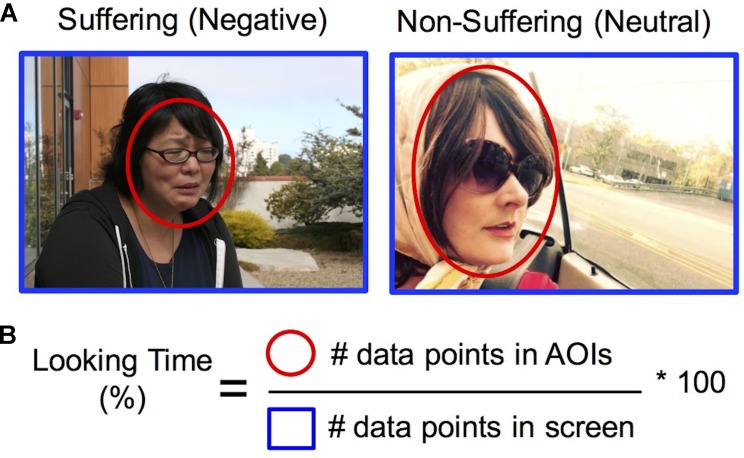
Quantifying visual attention to suffering. **(A)** Areas of interest (in red) were drawn around regions of the image depicting social and emotional information (see section “Materials and Methods” for full details). Images used for this figure represent examples of images that depict human suffering (Negative) or non-suffering (Neutral), which in the experiment were taken from the IAPS dataset (Lang et al., 1999). Persons depicted provided written consent for images to be used. **(B)** Looking time for each condition was computed by calculating the percentage of data points within the areas of interest (in red) compared to total data points in the entire screen (in blue). Visual preference for suffering was computed by a difference score for percentage looking time in Negative vs. Neutral AOIs, and training-related changes were computed from a difference score of Post–Pre training.

Eye-tracking data were analyzed to determine the amount of visual attention to social and emotion-relevant regions within the images. Looking Time was computed as the percentage of data points located within defined social and emotional areas of interest (AOIs) compared to total number of data points in the field of view within each 12-s trial (**Figure 1**) (van Reekum et al., 2007). Visual preference for suffering was computed by a difference score for percentage looking time in Negative–Neutral AOIs, and training-related changes were computed from a difference score of Post–Pre training. To determine the areas of interest (AOIs) on the images, two trained assistants independently drew (rectangular and elliptical) AOIs on each of the images using iView Analysis software (v. 1.09.29, SensoMotoric Instruments). An AOI was defined as object(s) within the image that provided social and affective meaning to the scene (e.g., the injured portions of a person’s body, faces within main characters in the scene, gun pointed at victim; see **Figure 1**). The AOIs generated by each individual were then compared. An AOI was only accepted when defined by both individuals, and when the size was within approximately 20% of the other. When these criteria were not met, a third person helped determine the AOIs size and placement. In-house software^2^ was then used to calculate the percentage of time looking within the AOIs compared to the entire image screen (**Figure 1**).

#### Altruistic Behavior

Altruistic behavior was assessed post-training with an economic decision-making task called the Redistribution Game, and previous results have found that compassion training increases costly redistribution compared to reappraisal training (Weng et al., 2013, 2015). Briefly, the Redistribution Game models both (1) unfair treatment of an anonymous stranger and (2) the participant’s willingness to redistribute funds at a personal cost. Using anonymous online interactions, participants first observed Player A (given $10) transfer an unfair amount of money ($1) to Player B who had no money. After witnessing this unequal exchange, participants could choose to spend any amount of their own money ($5) to redistribute funds from Player A to Player B (every $1 spent results in $2 redistributed from Player A to B). Participants were paid the amount that was left in their endowment after making the decision. Only participants who believed they were interacting with real participants and had valid visual attention data at both time points were included for exploratory data analyses.

#### Social Desirability

Positive social qualities such as compassion may be influenced by social desirability. In previous research with a similar sample, we found both self-reported compassion, as well as economic behavior in the Redistribution Game, were positively correlated with self-reported social desirability (Weng et al., 2013, 2015). We administered the Marlowe-Crowne Social Desirability (Crowne and Marlowe, 1960) scale to ensure that visual attention was not influenced by social desirability.

### Data Analysis

#### Overview

We analyzed our data from the Regulation condition in three main steps to identify group differences in (1) relationships between visual attention to suffering and neural responses (in the amygdala and whole-brain), (2) visual attention to suffering, and (3) relationships between visual attention to suffering and altruistic behavior. Because our *a priori* hypotheses concerned differences in visual attention to suffering *due to* compassion training, we first examined the data that reflected changes directly due to training (Post–Pre training contrast). In order to examine neural and visual responses to suffering (vs. non-suffering), the contrast of Negative–Neutral was used for all data analyses (e.g., visual preference for suffering). To understand the contribution of the Time factor in significant training-related effects, we performed follow-up tests in data obtained after training (Post-training) as well as before training (Pre-training). Finally, to examine whether results were specific to the Regulation condition, we performed parallel analyses in the Attend condition.

#### Relationships Between Visual Attention and fMRI Neural Responses (Group Level Analysis)

First, we investigated whether training-related changes in visual attention and neural responses to suffering differed between groups. We computed this using a Group (Compassion, Reappraisal) × Looking Time (continuous percentage looking time within AOIs) interaction test with the fMRI data (Negative–Neutral, Post–Pre training contrast in both measures), controlling for main effects of Group and Looking Time. We performed the interaction test within (1) a region of interest (ROI) of the bilateral amygdala and (2) the whole brain. To clarify the contributions of each time point to any significant interactions, we performed follow up interactions within each time point.

##### Region-of-interest (ROI) analyses within the amygdala

For the ROI analyses of the amygdala, we extracted average percent signal change from the left and right ROI masks at 50% probability from the Harvard-Oxford atlas in FSL (left amygdala ROI: 240 voxels or 1920 mm^3^; right amygdala ROI: 280 voxels or 2240 mm^3^; Desikan et al., 2006), and created summary bilateral amygdala scores for each time point as well as difference scores. We performed the interaction tests using IBM SPSS Statistics (version 24) using hierarchical linear regression on extracted PSC values from the bilateral amygdala. Main effect variables of Group and Looking Time were entered in Step 1, and the interaction variable of Group × Looking Time was entered in Step 2. In exploratory analyses, we examined potential hemispheric and subregional amygdala contributions by performing voxel-wise regression tests within the left and right amygdala (small-volume corrected within each hemisphere using Monte Carlo simulations, voxel-wise threshold at *p* < 0.05, corrected for multiple comparisons within the left and right amygdala at *p* < 0.05 and an extent of 416 and 464 mm^3^, respectively). The regression modeled the interaction of the Group × Looking Time on neural data, controlling for the main effects of Group and Looking Time.

##### Whole-brain analysis

A whole-brain voxel-wise interaction test of Group × Looking Time was conducted using 3dRegAna in AFNI, within functional data that were thresholded at 25% gray matter probability within the MNI152 atlas FSL, controlling for main effects of Group and Looking Time. The whole-brain analysis was corrected for multiple comparisons within the MNI152 atlas (thresholded at 25%) using Monte Carlo simulations (AlphaSim in AFNI; *p* < 0.01 corrected [cluster size ≥ 2496 mm^3^] after initial thresholding at *p* < 0.01 [*R*^2^ ≥ 0.291, *F*_3,21_ = 8.11]). To decompose the simple effects driving the interaction, regressions of looking time and neural activation were performed in each group, and significant voxels (*p* < 0.01) were identified within the significant interaction clusters. Result maps depict voxels from the significant (1) interaction cluster, (2) compassion simple effect, and (3) reappraisal simple effect (**Figure 3** and Supplementary Figure S1). Values were extracted only to determine the direction of the relationship and to inspect for outliers.

#### Visual Attention

To examine whether visual attention to suffering was impacted by compassion training, we computed a 3-way Group (COM, REP) × Time (Pre, Post) × Valence (Negative, Neutral) ANOVA on percentage looking time values when participants were employing their respective trainings. The main effects of Group, Time, and Valence, and the interactions of Group × Time, Group × Valence, Time × Valence, and Group × Time × Valence were modeled. Significant main effects and interactions were followed up with post-hoc testing. We performed a subsequent 3-way ANOVA with social desirability entered as a covariate to ensure any significant findings were not due to social desirability. Follow up tests within each time point were performed.

#### Visual Attention and Altruistic Behavior

Exploratory analyses were conducted to test whether greater visual attention to suffering after compassion training may reflect prosocial motivation and is associated with greater altruistic behavior. Analyses were conducted in a subset of participants who had both eye tracking data of adequate quality and valid redistribution responses (Compassion *n* = 8; REP *n* = 9, histograms displayed in Supplementary Figure S3). We investigated the relationship between looking time and redistribution behavior (calculated as rank values to decrease the influence of outliers; see Weng et al., 2013) using looking time metrics that reflected visual attention to suffering (Negative–Neutral contrast) across both time points and changes directly due to training (Post–Pre training). To gain a preliminary estimate of the direction and magnitude of relationship between visual attention and altruistic behavior, a Pearson’s correlation was computed between looking time and redistribution (rank) in each group. Using the Fisher r- to z-transformation, we also tested the difference in correlations between groups.

## Results

### Relationships Between Visual Attention and Neural Responses

#### Visual Attention and Amygdala Analyses

**FIGURE 2:**
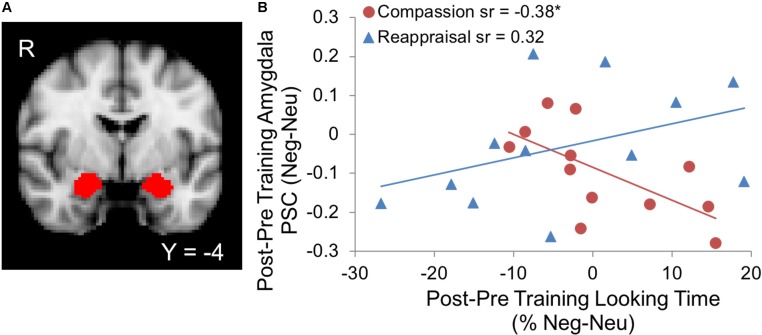
**(A)** Bilateral amygdala regions of interest (ROIs) from the Harvard-Oxford probabilistic atlas (Desikan et al., 2006) provided in FSL (thresholded at 50% probability and displayed on the 2 mm MNI152 atlas). **(B)** Increases in visual attention to suffering due to compassion training were associated with decreases in amygdala activation, whereas no relationship was found due to reappraisal training (Group × Looking Time interaction, *p* < 0.05; ^∗^Compassion simple effect *p* = 0.05). Both looking time percentage and amygdala activation values represent the contrast of suffering vs. non-suffering (Neg-Neu) and post vs. pre-training (Post–Pre). PSC denotes percent signal change of the BOLD response within the amygdala.

We examined how the training groups differ in their *relationship* between percentage looking time to suffering and amygdala activation given the amygdala’s involvement in motivational salience of stimuli (Cunningham and Brosch, 2012) and physiological responses in aversive emotional states (Davis and Whalen, 2001; Phelps and LeDoux, 2005). Visual attention was assessed using an eye-tracking metric of percentage looking time that calculated the percentage of data points within the social and emotion-relevant areas of interest (van Reekum et al., 2007), compared to data points in the entire image screen for images depicting suffering (negative) and non-suffering (neutral) (**Figure 1**). When we examined changes in visual attention to suffering and amygdala activation due to training (Post–Pre training, Negative–Neutral contrast in both metrics; L amygdala ROI size: 1920 mm3, R amygdala ROI size: 2240 mm3), we found that training group significantly modulated the association between amygdala activation and looking time (Group × Looking Time interaction Δ*R*^2^ = 0.24, *B* = -0.013, *SE* = 0.005, *F*_change_ = 6.91, *p* < 0.05, controlling for main effects of Group and Looking Time; **Figure 2**). In particular, the simple effects of the significant Group × Looking Time interaction revealed that increased visual preference to suffering produced by compassion training was associated with decreased activation in bilateral amygdala (*B* = -0.008, *SE* = 0.004, *t* = -2.05, *sr* = -0.38, *p* = 0.05), whereas the association between visual attention and amygdala activation due to reappraisal training was not significant (*B* = 0.004, *SE* = 0.003, *t* = 1.71, *sr* = 0.32, *p* = 0.10). Follow-up tests within each time point demonstrated that this interaction was primarily driven by the relationship between looking time and amygdala activation at post-training (*p* = 0.06) rather than at pre-training (*p* = 0.76). To investigate the laterality and potential subregions that may have contributed to this significant interaction, we performed exploratory voxel-wise analyses within the amygdala ROIs using the same interaction model. A cluster in the right amygdala survived correction (*p* < 0.05 within the right amygdala ROI) and was located in the dorsal aspect of the amygdala (Supplementary Figures S1A–C). Extracted values confirmed the direction of the relationship and were displayed for inspection purposes only (Supplementary Figure S1D). Analogous Group × Looking Time interaction tests in the Attend condition were not significant at Pre, Post, or Post-Pre training (*p*s > 0.32).

#### Visual Attention and Whole-Brain Analyses

**FIGURE 3:**
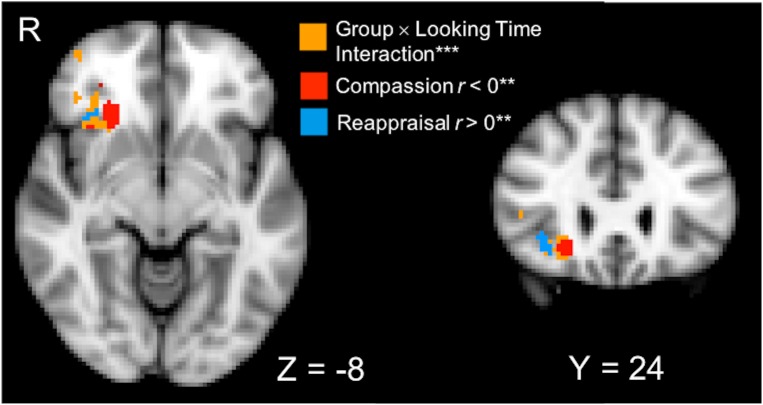
A cluster that spans the right anterior insula (AI) and orbitofrontal cortex (OFC) was identified from a whole-brain Group (Compassion, Reappraisal) × Looking Time (percentage) interaction performed on Post vs. Pre-training neural activation (PSC) in the Negative vs. Neutral conditions (orange voxels; ^∗∗∗^*p* < 0.01 voxel-wise threshold, *p* < 0.01 corrected, displayed on 2 mm MNI template). To characterize the simple effects within the significant interaction voxels, regression tests of Looking Time and neural activation (Negative–Neutral) were performed in each training group (^∗∗^ thresholded at *p* < 0.01). Within the significant interaction cluster, red voxels indicate that the Compassion group showed a significant negative relationship between looking time and neural activation, and blue voxels indicate that the Reappraisal group showed a positive relationship between looking time and neural activation.

We hypothesized that compassion training-related changes in visual attention to suffering would be differentially associated with changes in neural responses to human suffering compared to reappraisal training (Post–Pre training, Negative–Neutral contrast). The whole-brain Group × Looking Time interaction test on training-related neural responses to suffering revealed a significant cluster spanning the right anterior insula (AI) and orbitofrontal cortex (OFC; 4096 mm^3^, MNI peak voxel: [24, 26, -8]; *p* < 0.01, corrected; **Figure 3**]. To characterize the direction of the interaction in the right AI/OFC, simple effects regression testing within the cluster showed that changes due to compassion training resulted in a negative relationship between visual attention and brain activation to suffering (voxels thresholded at *p* < 0.01 depicted in red in **Figure 3**), while changes due to reappraisal training resulted in a positive relationship between visual attention and brain activation (voxels thresholded at *p* < 0.01 depicted in blue in **Figure 3**). Because associations with looking time and amygdala activation were driven by post-training data, we also report the results of the whole-brain interaction in post-training data only (see Supplementary Material and Supplementary Figure S2).

### Visual Attention

**FIGURE 4:**
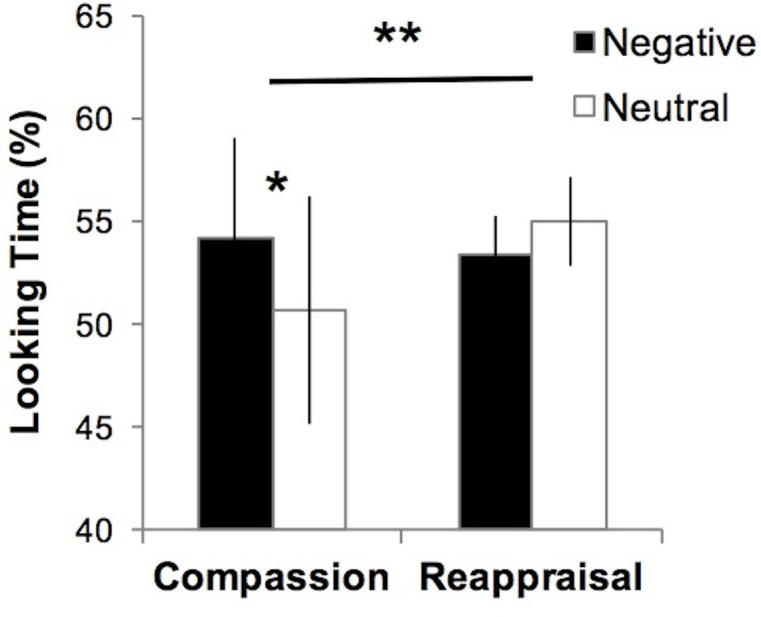
Participants who employ compassion show greater visual preference for suffering, where visual attention to emotional regions of images depicting human suffering (Negative) was greater compared to non-suffering (Neutral). Those who employ reappraisal show the opposite pattern at a non-significant level (^∗∗^Group × Valence interaction test *F*_1,22_ = 8.47, *p* < 0.01; ^∗^Paired *t*-test, *p* < 0.05). No group differences were found for Negative or Neutral images (*p*s > 0.47). Error bars indicate standard error of the mean.

We tested whether visual attention to suffering was impacted by compassion training using an eye-tracking metric of percentage looking time that calculated the percentage of data points within the social and emotion-relevant areas of interest (van Reekum et al., 2007), compared to data points in the entire image screen for images depicting suffering (negative) and non-suffering (neutral) (**Figure 1**). A 3-way ANOVA of Group (Compassion, Reappraisal) × Time (Pre-training, Post-training) × Valence (Negative, Neutral) on the eye-tracking data yielded a significant Group × Valence interaction (*F*_1,22_ = 8.47, *p* < 0.01; **Figure 4**). This Group × Valence interaction remained significant when controlling for self-reported social desirability (*F*_1,21_ = 8.14, *p* < 0.01). Upon investigating the drivers of this interaction, we found that across both time points, the compassion group spent more time looking at social and emotional regions of negative images compared to neutral images (Negative mean = 54.18, SD = 16.79; Neutral mean = 50.70, SD = 18.98; *t*_11_ = 2.85, *p* < 0.05; **Figure 4**), whereas the reappraisal group did not (Negative mean = 53.33, SD = 6.93; Neutral = 55.00, SD = 7.32, *t*_11_ = -1.31, *p* = 0.22). Within each valence, the groups did not significantly differ in looking time (*p*s > 0.47). In summary, employing compassion (compared to reappraisal) produced a visual preference for cues of suffering compared to non-suffering across both time points. No other main effects or interactions were found within the 3-way ANOVA in the Regulation condition, and the Attend condition did not yield a significant 3-way interaction or 2-way Group × Valence interaction (which was present in Regulation trials; *p*s > 0.34).

### Exploratory Analyses: Visual Attention and Altruistic Behavior

In exploratory analyses in a subset of participants with valid prosocial behavioral data, we investigated whether greater visual attention to suffering (Negative–Neutral contrast) was associated with altruistic behavior as measured by the redistribution game after training. Indeed, the compassion group showed a trend towards a positive association between visual attention to suffering and prosocial behavior whereas the reappraisal group showed a negative association (slope difference, *z* = 1.62, *p* = 0.10; Supplementary Figure S3). Due to the early exploratory nature of the analyses, these results are further reported in the Supplementary Material to aid future hypotheses and research.

## Discussion

In summary, the present findings, whilst preliminary due to the small sample size, suggest that after compassion meditation training, individuals who visually engage more with suffering while actively generating compassion show *decreased* activation in the amygdala and right AI/OFC, brain regions sensitive to processing negatively valenced and arousing stimuli. This is in contrast to individuals who engage in reappraisal training (which cultivates cognitive reinterpretation of stimuli to decrease personal negative emotions), where greater visual preference for suffering due to training was not associated with amygdala activation, and was positively associated with activation in AI/OFC. Importantly, these associations were found specifically when participants were engaging in compassion (vs. reappraisal) as a regulatory means to change emotional responses, and not when they were simply attending to images of suffering. Given the low number of participants, these should be considered preliminary results that should be interpreted with caution and confirmed with future research containing larger sample sizes. We tentatively interpret the results and provide suggestions for future research.

Together, these findings may suggest that compassion training may support greater visual engagement with suffering (vs. non-suffering) while mitigating neural responses to aversive events that may activate the sympathetic nervous system and inhibit prosocial behavior, therefore cultivating a more balanced relationship with stimuli of suffering. When examining visual attention behavior independently of neural responses, participants engaging in compassion (across both time points) spent more time visually attending to cues of suffering compared to non-suffering relative to those who engage in reappraisal. This difference in looking time by valence was specific to compassion – when participants engaged in reappraisal, there was no difference in the amount of the time they visually attend to suffering vs. non-suffering. Of note, these groups did not differ in absolute looking time in either valence alone, suggesting that the interaction is driven by the within-compassion group difference of visual attention to suffering vs. non-suffering (e.g., visual preference for suffering).

We found that compassion training-related *increases* in visual attention to suffering were associated with training-related *decreases* in amygdala activation, a brain region involved in processing motivational salience, which include stimuli that are negative, positive, arousing, interesting, or unusual, depending on a perceiver’s goals (Adolphs et al., 1999; Hamann et al., 2002; Phan et al., 2003; Cunningham and Brosch, 2012). Exploratory voxel-wise analyses highlighted a cluster in the right dorsal amygdala primarily contributing to this effect. The dorsal amygdala has been implicated in processing arousal of social stimuli (Davis et al., 2010), and may include the centromedial nucleus of the amygdala, which has direct projections to brain regions that mediate sympathetic physiological responses typical of fear and anxiety such as changes in blood pressure, heart rate, and endocrine responses (Davis and Whalen, 2001; Phelps and LeDoux, 2005). Therefore, decreased activation in this region after compassion training associated with greater visual attention may reflect decreased arousal in response to suffering, which may mitigate empathic distress and facilitate prosocial engagement (Batson, 1991).

Furthermore, this finding was driven by relationships found at post- and not pre-training, further suggesting that this outcome may be specifically due to compassion training. This suggests that the compassion trainees who were able to visually engage more with cues of suffering were doing so despite not further engaging neural regions typically sensitive to valence and arousal content of negative stimuli. It is possible that these participants, despite allocating attention to aversive visual cues may be less reactive to suffering, and could potentially show less aversive psychophysiological responses in skin conductance and heart rate which is partially mediated by the amygdala. Disambiguating the directionality of this –i.e., determining whether visual behavior change influences neural changes or vice versa—is a critical direction for future research. In addition, future work should examine whether a more calm, balanced attention to human suffering (Halifax, 2012) characterized by attenuated sympathetic-nervous system responding causally supports prosocial behavior.

It is important to note as the field is moving beyond the negative emotion-centric interpretations of amygdala engagement (LeDoux and Hofmann, 2018), amygdala functioning has also been recognized to be generally involved in motivational salience and be sensitive to not only negative but also positive stimuli, arousal, and novelty (Cunningham and Brosch, 2012). Although it is likely that compassion training enhances the motivational salience of suffering, the negative relationship between visual preference for suffering and amygdala in our dataset suggest that amygdala in this study paradigm more likely reflected *decreased* aversive responses to suffering rather than increased motivational salience. Although other studies have not specifically investigated the relationship between visual attention and amygdala responses due to compassion meditation, the direction of the amygdala finding is supported by research in expert meditation practitioners showing decreased amygdala responses to film clips of suffering while engaging in compassion vs. attending (Engen and Singer, 2015). However, this finding is in contrast to previously reported findings by Desbordes et al. (2012), where they showed greater amygdala activation to negative stimuli was increased after compassion training, albeit in an “attend” state rather than an active compassion state. One putatively important difference between the present study and Desbordes et al. (2012) is that our study had a shorter training period (7 h total over 2 weeks compared to 8 weeks), and perhaps changes in amygdala function early on in training while actively engaging in compassion reflect reduced aversive responses to suffering. Alternatively, our finding may also reflect more sustained amygdala activation over the length of the entire trial (modeled by a block function over 12s), which we modeled to associate with looking time over the entire trial. It is plausible that motivational salience may be better modeled by a different amygdala response function focused on initial phasic responses, and future fMRI research should tease these different functional aspects of neural activation apart by using multivariate analysis combined with subjective reports of salience, empathic concern, and empathic distress (e.g., Ashar et al., 2017).

After compassion training, greater visual preference for suffering was negatively associated with activation in the right AI and OFC; whereas after reappraisal training, visual attention to suffering was associated with greater AI/OFC activation. The AI has been consistently implicated in studies and meta-analyses of empathy (Lamm et al., 2011) and is thought to be involved in representing empathic distress. Specifically, the AI is considered part of the shared representations model of empathy (Zaki and Ochsner, 2012), where the perceiver of suffering may show activation in that region when they both experience and observe situations. The negative relationship between visual preference for suffering and AI may indicate that compassion trainees are able to visually engage more with suffering while dampening their shared representation of empathic distress.

The OFC is known to be bidirectionally connected with the amygdala (Salzman and Fusi, 2010), and from a decision-making model of how sensory stimuli are processed into choices, the amygdala and OFC and are part of the neural circuitry that represent the affective value of stimuli (Grabenhorst and Rolls, 2011). These regions have both been previously implicated in the encoding value and valence of a variety of emotional stimuli across different sensory modalities (Grabenhorst and Rolls, 2011). From this framework, the negative relationship between visual attention to suffering and activation in the amygdala and OFC after compassion training may reflect that attention to negative stimuli is being decoupled with neural responses that are typically activated in response to aversive stimuli. This may suggest that compassion trainees may be learning to visually engage more with suffering while cultivating a more equanimous emotional response (Desbordes et al., 2014). In reappraisal trainees, greater visual attention to suffering may be associated with greater AI/OFC activation because those who visually attended to suffering may have been less successful at implementing reappraisal strategies and their associated attention mechanisms (van Reekum et al., 2007), which typically entail distancing from or reinterpreting the emotionally challenging stimulus.

We suggest that employing compassion in general may result in a visual preference for cues of suffering, where greater visual attention is directed to cues of suffering than neutral stimuli. This visual preference for cues of suffering may be due to the prosocial motives of compassion to care for and help those who are suffering (Isaacowitz, 2006), which may result in heightened visual attentiveness to understand the context of suffering, along with the display of non-verbal cues which signals attentiveness to those in need (Ellsworth and Ludwig, 1972). The reappraisal group did not demonstrate this valence-based visual preference. The implemented reappraisal strategies may have been sufficient to regulate negative emotion, without gaze aversion being needed to further decrease negative emotion (van Reekum et al., 2007), particularly because the negative images used in the study were not extremely intense. Comparing the groups within each valence, engaging in compassion vs. reappraisal did not result in significant differences in looking time. This suggests that employing compassion and reappraisal result in a similar absolute looking time to visual cues of suffering as well as non-suffering; however, the relative looking time (e.g., visual preference) to suffering vs. non-suffering is more pronounced when employing compassion. Future studies that are more highly powered should further investigate this question.

Counter to our hypotheses, we observed changes in visual attention to suffering as a function of engaging in compassion in general (across both time points), as opposed to directly due to compassion training (i.e., training-related changes). We interpret the lack of training-level changes with caution because of the short duration of training and the relatively small sample size—therefore, future research should investigate these questions with longer training length as well as larger sample sizes. Although compassion meditation did not directly increase visual attention to suffering, tests employing an individual differences model suggested that only compassion *training-related* changes in visual attention to suffering were related to differential neural responses. In addition, early exploratory analyses associated visual preference for suffering due to compassion training with greater prosocial responses; however, this trend-level finding should be interpreted with caution and is reported for completeness and to inform future research. Future work should collect larger samples with high quality eye tracking data, and should include a “No Training” control group with an Attend condition that is not intermixed with Regulation conditions, which would provide a better assessment of baseline visual attention and neural responses. Because the reappraisal training condition also actively regulates emotions and changes eye gaze behavior, direct comparison of eye gaze behavior between reappraisal and compassion groups is challenging to readily interpret. Finally, the fMRI paradigm could be improved by increasing the number of trials, as well as balancing the number of trials, within each valence.

In summary, the current findings, whilst preliminary due to the low sample size, suggest that engaging in compassion as an emotion regulation strategy towards suffering resulted in increases in visual attention to regions of images depicting suffering compared to non-suffering. Thus, engaging in compassion may be a useful strategy in situations where attention needs to turn toward suffering, such as when a doctor aids a patient. While our results suggest that compassion training may not be needed to produce this visual change, individual differences in greater visual preference for suffering due to compassion meditation training may help decrease aversive neural responses to suffering, even as visual attention to suffering increases. This may help individuals stay calm in the face of suffering and more readily able to engage in prosocial action. Further research should explore these questions with larger sample sizes, and determine whether compassion intervention strategies could aid people who are often exposed to others’ suffering such as health care workers and caregivers.

## Author Contributions

HW, DS, GR, and RD designed the study. HW and DS collected data. HW, RL, GR, and RD conceived data analyses. HW analyzed data. All authors contributed to writing the manuscript.

## Conflict of Interest Statement

RD serves on the board of directors for the following non-profit organizations: Healthy Minds Innovations and the Mind and Life Institute. HW serves as a Fellow for the Mind and Life Institute. No donors, either anonymous or identified, have participated in the design, conduct, or reporting of research results in this manuscript. The other authors declare that the research was conducted in the absence of any commercial or financial relationships that could be construed as a potential conflict of interest.

## Footnotes

1 https://web.archive.org/web/20150925171213/http://brainimaging.waisman.wisc.edu/~jjo/fieldmap_correction/fieldmap_correction.html

2 https://github.com/njvack/gazehound
