# Supplementary material for: Visual Attention to Suffering After Compassion Training Is Associated With Decreased Amygdala Responses
Source: Front Psychol. 2018 May 22;9:771. doi: 10.3389/fpsyg.2018.00771 (PMC5972817; doi:10.3389/fpsyg.2018.00771)
Supplement: Supplementary file 1 [file Data_Sheet_1.docx]

***Supplementary Material***

**Visual attention to suffering after compassion training is associated with**

**decreased amygdala responses**

Helen Y. Weng*, Regina Lapate, Diane E. Stodola, Gregory M. Rogers

& Richard J. Davidson

*** Correspondence:** Helen Weng: Helen.Weng@ucsf.edu

**Supplementary Data**

**Visual attention.** The main finding in the visual attention data was a significant Group × Valence interaction, where the compassion group visually attended to cues of suffering more than non-suffering compared to reappraisal, across both time points. Due to our *a priori* hypotheses that visual attention to suffering would be more pronounced after compassion training, we examined the simple effects driving the Group × Valence interaction within each time point and found that it was driven primarily by visual attention at post-training (*F*_1,22_ = 5.80, *p* < 0.05) vs. at pre-training (*F*_1,22_ = 0.96, *p* = 0.34).

**Data analyses across both time points.** We also performed exploratory follow-up tests in data that reflected generally engaging in compassion vs. reappraisal (i.e. across both time points), to follow up on the main finding from analytic step 2 (see Results in main manuscript).

**Visual attention and neural responses.** Based on the main findings within the eye-tracking data alone (see Results and **Fig. 4**), we investigated whether groups across both time points differed in their relationship between visual attention to suffering and neural responses. We computed this using a Group (Compassion, Reappraisal) × Looking Time interaction test with the average scores of fMRI data across both time points (Negative–Neutral contrast in both measures), controlling for main effects of Group and Looking Time. Employing compassion vs. reappraisal in general (i.e. across both time points) did not yield significant associations between visual attention to suffering and bilateral amygdala activation (Negative–Neutral contrast in both metrics; Group × Looking Time interaction *p* = 0.42). No significant clusters were found in the whole-brain Group × Looking Time interaction tests conducted across both time points.

**Whole-brain analysis of visual attention and neural responses to suffering at post-training.**

**Method**

A whole-brain voxelwise interaction test of Group × Looking Time was conducted using 3dRegAna in AFNI on post-training PSC data (Negative-Neutral contrast) in functional data that were thresholded within the MNI152 atlas (thresholded at 25%) in FSL. The whole-brain analysis was corrected for multiple comparisons within the MNI152 atlas (thresholded at 25%) using Monte Carlo simulations (AlphaSim in AFNI; *p* < .01 corrected after initial thresholding at *p* < 0.01). To characterize the simple effects within the significant interaction clusters, regression tests using 3dRegana were conducted within each group. The regressions tested the relationship between Looking Time and PSC within each significant cluster. Voxels from the regressions that survived a *p* < 0.01 threshold were inspected for the direction of the association (positive values indicate a positive relationship, negative values indicate a negative relationship) and are displayed in **Fig. S2** for interpretation purposes only.

**Results and Discussion**

We hypothesized that individual differences in visual attention to suffering after compassion vs. reappraisal training would be associated with differences in neural activation in response to human suffering (vs. non-suffering; Negative vs. Neutral contrast). The whole-brain Group × Looking Time interaction test on neural responses to suffering at post-training revealed significant clusters within the right orbitofrontal cortex (OFC; 5672 mm^3^, MNI peak voxel: [20, 44, -14], center of mass: [33, 35, -11]; *p* < 0.01, corrected; **Fig. S2A**] and pregenual anterior cingulate cortex (pgACC; 4288 mm^3^, MNI peak voxel: [8, 34, 12]; *p* < 0.01, corrected; **Fig. S2B**). Both clusters were corrected for multiple comparisons at *p* < 0.01 within the MNI 25% probability atlas (extent of 312 voxels or 2496 mm^3^) after a voxelwise threshold of *R*^2^ ≥ 0.291, *F_3,21_* = 8.11, *p* < 0.01. The pgACC cluster spanned both left and right hemispheres.

To characterize the direction of the interaction, simple effects regression testing within both clusters showed that the compassion group had a negative relationship between visual attention and brain activation to suffering (voxels thresholded at *p* < 0.01 depicted in red in **Fig. S2**), while the reappraisal group had a positive relationship between visual attention and brain activation in the right OFC (voxels thresholded at *p* < 0.01 depicted in blue in **Fig. S2**).

After compassion training, greater visual attention to suffering vs. non-suffering was associated with less activation in the right OFC, which is known to be bidirectionally connected with the amygdala (Salzman and Fusi, 2010), as well as in the pgACC (in a subset of voxels); whereas after reappraisal training, greater visual attention to suffering vs. non-suffering was associated with greater activation in the right OFC. From a decision-making model of how sensory stimuli are processed into choices, the amygdala, OFC, and pgACC are part of the neural circuitry that represent the affective value of stimuli (Grabenhorst and Rolls, 2011). These regions have both been previously implicated in the encoding value and valence of a variety of emotional stimuli across different sensory modalities^2^. From this framework, the negative relationship between visual attention to suffering and activation in the OFC and pgACC after compassion training may reflect that attention to negative stimuli is being decoupled with neural responses that are typically elicited in response to aversive stimuli. This suggests that compassion trainees may be learning to visually engage more with suffering while cultivating a more balanced emotional response.

**Exploratory analyses: visual attention and altruistic behavior.**

Previous research demonstrated that compassion meditation increases altruistic redistribution compared to reappraisal training (Weng et al., 2013). In exploratory analyses in a subset of participants with valid behavioral data (Compassion n = 8, Reappraisal n = 9), we investigated whether greater visual attention to suffering (Negative-Neutral contrast) was associated with altruistic behavior as measured by the redistribution game after training. Ranks of raw redistribution values were used to decrease the influence of outliers (ranked out of 41 participants reported in Weng et al., 2013). Because of the small sample sizes, histograms of ranked Redistribution responses for the Compassion and Reappraisal training groups are displayed in **Fig. S3A-B**. In preliminary examinations of the potential relationship between visual attention to suffering and subsequent altruistic behavior in a separate task, correlations between visual attention data and altruistic behavior were performed in each group, and the difference between correlations between groups was also tested.

When examining whether *changes* in visual attention to suffering due to training (Post-Pre training contrast) were associated with altruistic redistribution, we found a trend-level difference between training groups (*z* = 1.62, *p* = 0.10), where increased visual attention to suffering while regulating was positively associated with altruistic behavior towards a stranger due to compassion training at a non-significant level (*r*_7_ = 0.41, *p* = 0.31; **Fig. S3C**), and negatively associated with altruistic behavior due to reappraisal training at a non-significant level (*r*_8_ = -0.50, *p* = 0.18; **Fig. S3D**). This trend may be driven by the relationship between visual attention at post-training and redistribution, where a similar near-trend level result was found between groups (Post-training *z* = 1.40, *p* = 0.13; Pre-training *z* = -1.05, *p* = 0.15), and a similar non-significant association was observed in each group (Compassion *r*_7_ = 0.40, Reappraisal *r*_8_ = -0.45). Correlations in the Attend condition were not significant (Compassion *r*_7_ = -0.59, *p* = 0.12; Reappraisal *r*_8_ = 0.19, *p* = 0.63, and the difference between correlations was not significant (*z* = -1.44, *p* = 0.15).

We found that the groups marginally differ in their relationship between training-related changes in visual attention to suffering and redistribution behavior. Due to small sample sizes, we tentatively interpret the initial pattern of results to aid future research hypotheses and study design. Increases in visual attention to suffering due to compassion training were associated with greater prosocial behavior at a non-significant level, which may suggest that greater visual attention to suffering reflects a prosocial motive (rather than other potential motives such as *schadenfreude* or enjoying another’s suffering). In contrast, increases in visual attention to suffering due to reappraisal training were associated with less prosocial behavior (at a non-significant level), which suggests that visual attention to suffering may not reflect prosocial motives, and may be related to less successful reappraisal. Given the small sample, however, these results should be considered preliminary, and future studies should further investigate the relationship between visual behavior towards suffering and prosocial behavior within compassion training.

1. **Supplementary Figures**

**
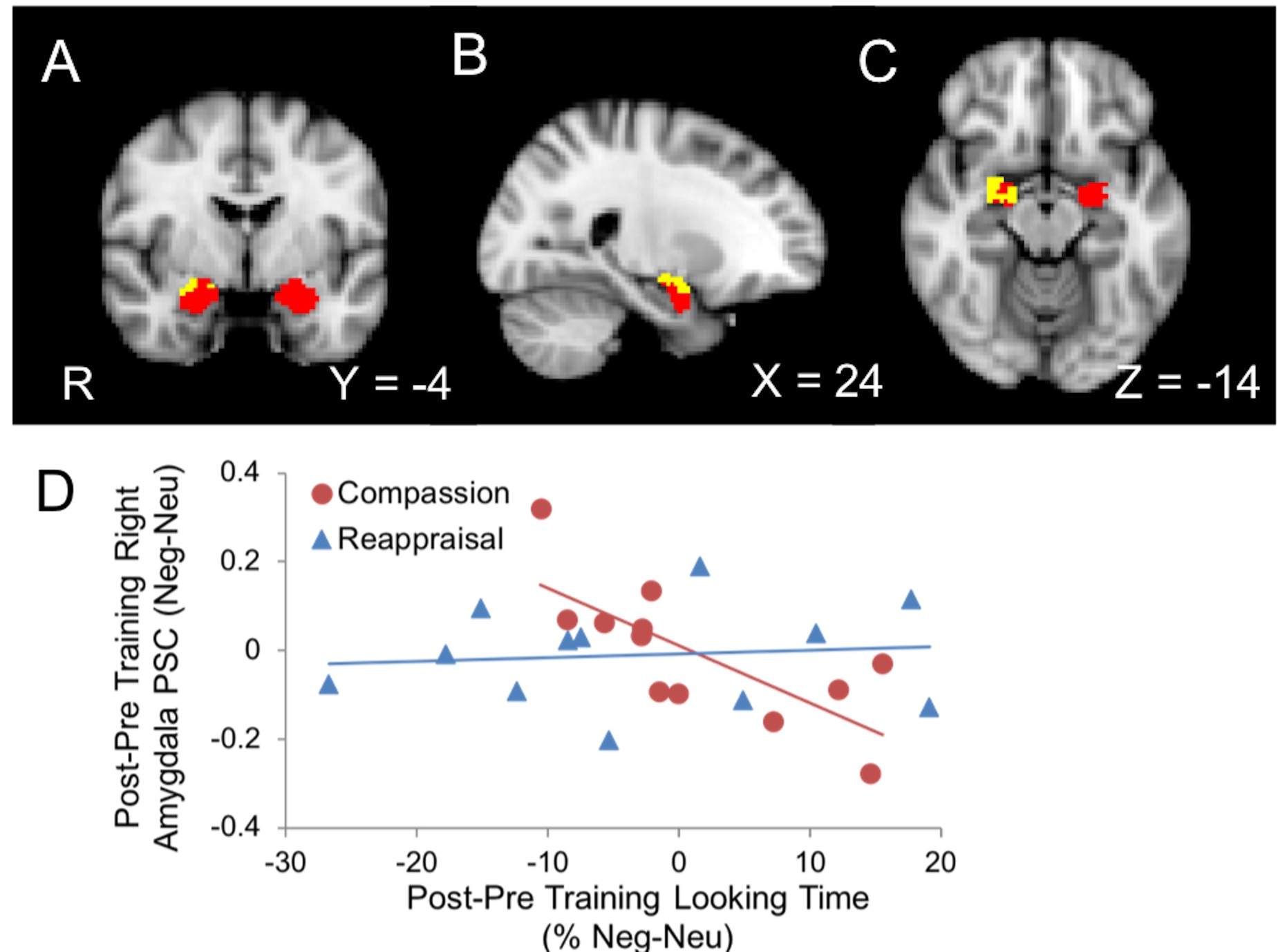
**

**Fig. S1.** Significant interaction cluster within the right amygdala from **A)** coronal, **B)** sagittal, and **C)** axial views displayed on the 2mm MNI152 template. Red voxels indicate the amygdala ROIs from the Harvard-Oxford atlas (50% gray matter probability, Desikan et al., 2006), and yellow voxels indicate the significant cluster (voxel-wise threshold at *p* < 0.05, corrected for multiple comparisons within the right amygdala ROI at *p* < 0.05 and an extent of 464 mm^3^). The cluster was identified from a voxel-wise interaction test of Group (Compassion, Reappraisal) × Looking Time (percentage) interaction performed on Post-Pre training neural activation (PSC) in the Negative vs. Neutral conditions (controlling for main effects of Group and Looking Time). The cluster is located on the dorsal portion of the right amygdala (cluster size: 576 mm^3^, peak voxel: 22, 2, -18 [*R*^2^ = 0.50]). **D)** Extracted values from this cluster were displayed to confirm the direction of the relationship, where the Compassion group shows that training-related increases in visual preference for suffering were associated with training-related decreases in amygdala activation to suffering.


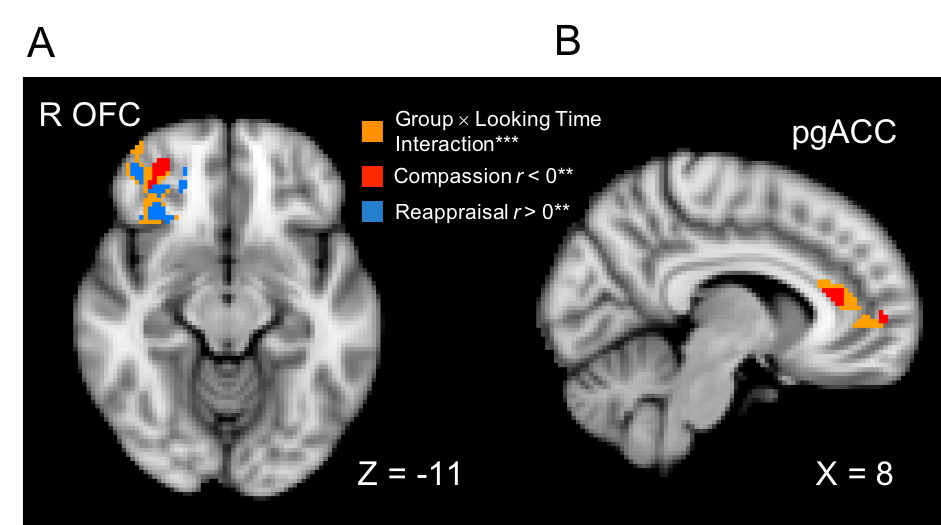


**Figure S2.** Clusters in the **(A)** right orbitofrontal cortex (R OFC) and **(B)** pregenual anterior cingulate cortex (pgACC) were identified with a whole-brain Group (Compassion, Reappraisal) × Looking Time (percentage) interaction performed on post-training neural activation (PSC) in the Negative vs. Neutral conditions (orange voxels; *** *p* < 0.01 voxelwise threshold, *p* < 0.01 corrected within the MNI 25% gray matter probability mask). To characterize the simple effects within the significant interaction voxels, regression tests of Looking Time and neural activation (Negative-Neutral) were performed in each training group (** thresholded at *p* < 0.01). Red voxels indicate that within the R OFC and pgACC, the Compassion group shows a significant negative relationship between looking time and neural activation. Blue voxels indicate that within the R OFC, the Reappraisal group shows a positive relationship between looking time and neural activation. Maps are displayed on the 2mm MNI152 template provided in FSL.

**
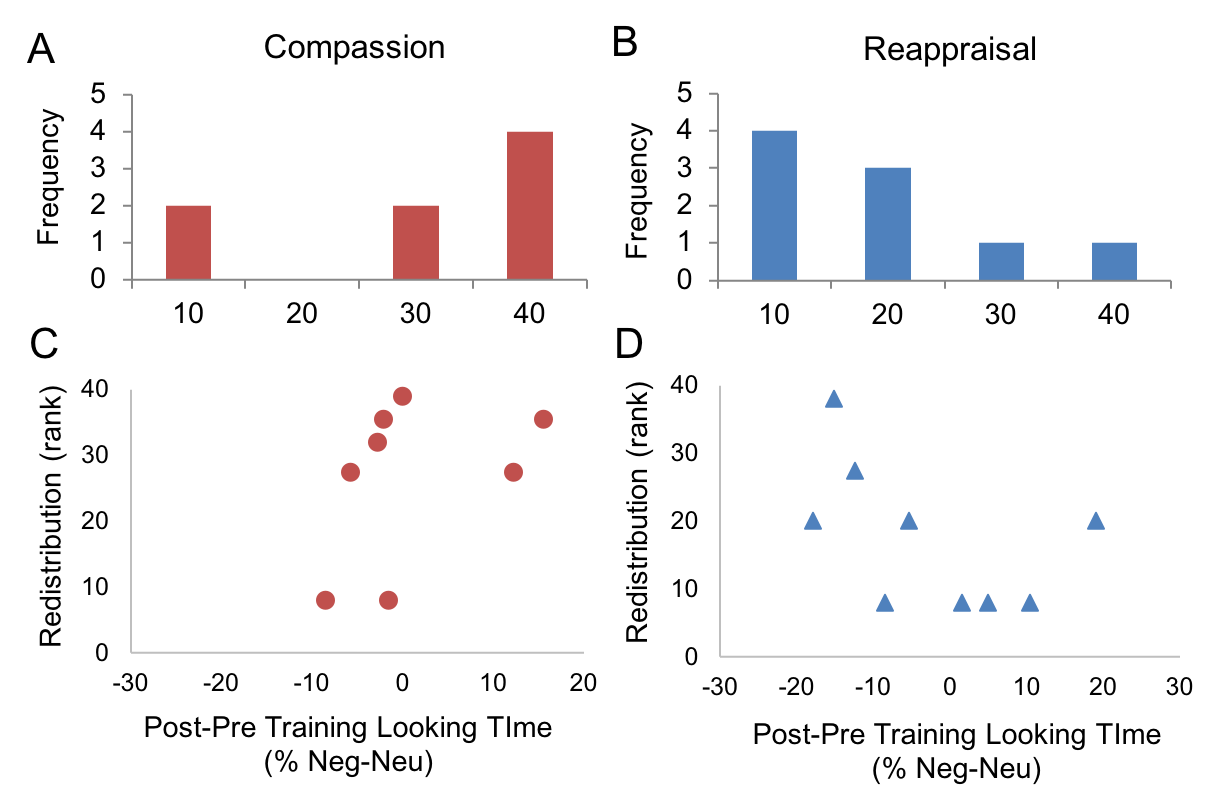
**

**Figure S3.** Exploratory analyses in a subset of participants (Compassion n = 8, Reappraisal n = 9) investigated the relationship between visual attention to suffering due to compassion and reappraisal training and subsequent altruistic behavior in the Redistribution Game. Histograms of redistribution behavior after **A)** Compassion and **B)** Reappraisal training, ranked out of 41 participants from Weng et al. (2013). Histograms represent bins of 10 (0-10, 11-20, 21-30, 31-40). The lowest rank of 8 indicates spending $0/$5 in the Redistribution Game, a rank of 20 indicates spending $0.50 and redistributing $1 from the dictator to the victim, and the highest rank of 39 in this sample indicates spending $2.50 and redistributing $5 from the dictator to the victim. Scatterplots depicting the putative relationship between changes in visual attention to suffering and altruistic redistribution behavior due to **C)** Compassion training (*r*_7_ = 0.41, *p* = 0.31) and **D)** Reappraisal training (*r*_8_ = -0.50, *p* = 0.18). The difference between correlations was trend level (*p* = 0.10). Looking time percentage was computed from a difference score of Post-Pre training and Negative-Neutral images.

**References**

Desikan, R. S., Ségonne, F., Fischl, B., Quinn, B. T., Dickerson, B. C., Blacker, D., et al. (2006). An automated labeling system for subdividing the human cerebral cortex on MRI scans into gyral based regions of interest. *NeuroImage* 31, 968–980. doi:10.1016/j.neuroimage.2006.01.021.

Grabenhorst, F., and Rolls, E. T. (2011). Value, pleasure and choice in the ventral prefrontal cortex. *Trends Cogn. Sci.* 15, 56–67. doi:10.1016/j.tics.2010.12.004.

Salzman, C. D., and Fusi, S. (2010). Emotion, Cognition, and Mental State Representation in Amygdala and Prefrontal Cortex. *Annu. Rev. Neurosci.* 33, 173–202. doi:10.1146/annurev.neuro.051508.135256.

Weng, H. Y., Fox, A. S., Shackman, A. J., Stodola, D. E., Caldwell, J. Z. K., Olson, M. C., et al. (2013). Compassion training alters altruism and neural responses to suffering. *Psychol. Sci.* 24, 1171–1180. doi:10.1177/0956797612469537.
